# Peculiarities of Yeasts and Human Telomerase RNAs Processing

**Published:** 2016

**Authors:** M.P. Rubtsova, D.P. Vasilkova, Yu.V. Naraykina, O.A. Dontsova

**Affiliations:** Lomonosov Moscow State University, Chemistry Department, Leninskie gory, 1, bld. 3, Moscow, 119991 , Russia; Lomonosov Moscow State University, Belozersky Institute of physico-chemical biology, Leninskie gory, 1, bld. 40, Moscow, 119991, Russia; Skolkovo Institute of Science and Technology, Skolkovo Innovation Center, bld. 3, Moscow, 143026 , Russia; Lomonosov Moscow State University, Faculty of bioengineering and bioinformatics, Leninskie gory, 1, bld. 73, Moscow, 119991, Russia

**Keywords:** exosome, processing, splicing telomerase, telomerase RNA, transcription

## Abstract

Telomerase is one of the major components of the telomeres –– linear eukaryotic chromosome ends – maintenance system. Linear chromosomes are shortened during each cell division due to the removal of the primer used for DNA replication. Special repeated telomere sequences at the very ends of linear chromosomes prevent the deletion of genome information caused by primer removal. Telomeres are shortened at each replication round until it becomes critically short and is no longer able to protect the chromosome in somatic cells. At this stage, a cell undergoes a crisis and usually dies. Rare cases result in telomerase activation, and the cell gains unlimited proliferative capacity. Special types of cells, such as stem, germ, embryonic cells and cells from tissues with a high proliferative potential, maintain their telomerase activity indefinitely. The telomerase is inactive in the majority of somatic cells. Telomerase activity *in vitro *requires two key components: telomerase reverse transcriptase and telomerase RNA. In cancer cells, telomerase reactivates due to the expression of the reverse transcriptase gene. Telomerase RNA expresses constitutively in the majority of human cells. This fact suggests that there are alternative functions to telomerase RNA that are unknown at the moment. In this manuscript, we review the biogenesis of yeasts and human telomerase RNAs thanks to breakthroughs achieved in research on telomerase RNA processing by different yeasts species and humans in the last several years.

## INTRODUCTION

Telomerase is a ribonucleoprotein complex comprising a reverse transcriptase (TERT) – a protein subunit enabling polymerase activity, and telomerase RNA (TER) [1, 2]. Telomerase RNA contains a template for the synthesis of telomeres and has an important architectural function: it acts as a structural framework for the formation of the active enzyme [3]. Different elements of the complex spatial structure of telomerase RNA are involved in the formation of the active telomerase center, promoting the effective addition of nucleotides during the synthesis of a telomeric repeat, as well as the translocation of the enzyme at the telomere required for the processive synthesis of a long telomeric sequence [4]. Additional protein factors interact with different domains of telomerase RNA and are necessary for its stabilization, efficient assembly, and the regulation of enzyme activity, localization, and transport within the cell.

## STRUCTURE OF TELOMERASE RNA

**Fig. 1:**
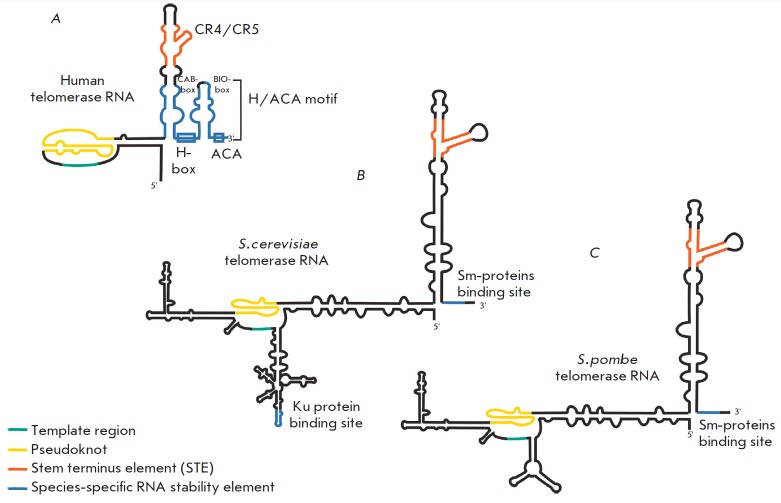
Structures of yeasts and human telomerase RNAs. A. Schematic model of human telomerase RNA secondary structure. B. Schematic model of *S.cerevisiae *telomerase RNA secondary structure. C. Schematic model of *S.pombe *telomerase RNA secondary structure.

Despite the high degree of variation in term of their sizes and nucleotide sequences, telomerase RNAs in yeast and mammals share four conserved structural elements necessary for the formation and functioning of the enzyme [5-11]. The template region, as its name implies, serves as a template for telomere synthesis [3], pseudoknot is involved in the positioning of the template region in the active site of the enzyme [12], and together with the STE-element (stem-terminus element) it interacts with TERT, whereas the species-specific 3’-terminal element ensures the stability of telomerase RNA [13] and is required for its proper intracellular localization [14-16] (*Fig. 1*).

## PROCESSING AND LOCALIZATION OF TELOMERASE RNA

**Processing and localization of telomerase RNA of Saccharomyces cerevisiae (TLC1)**

In the process of RNA transcription, polymerase II synthesizes two forms of telomerase RNA: a long polyadenylated one and a short non-polyadenylated one. The fate of the long polyadenylated form is poorly understood at the moment. It is known that this form accounts for 10% of the total telomerase RNA in a cell, but it is not associated with the active telomerase [17]. It is assumed that the long polyadenylated telomerase RNA can be processed to the “mature” catalytically active form. The expression of TLC1 in *S. cerevisiae *yeast is known to be regulated by a strong promoter (pGal4), which directs the expression of protein-encoding genes and leads to the accumulation of the polyadenylated form, but it does not affect the content of the non-polyadenylated one, whereas disruption of the polyadenylation system prevents the formation of the polyadenylated form and greatly reduces the content of “mature” TLC1 in a cell [17, 18]. These data suggest that the long polyadenylated primary transcript may undergo processing to yield mature telomerase RNA, although there are no experimental data to support such a mechanism yet.

**Fig. 2:**
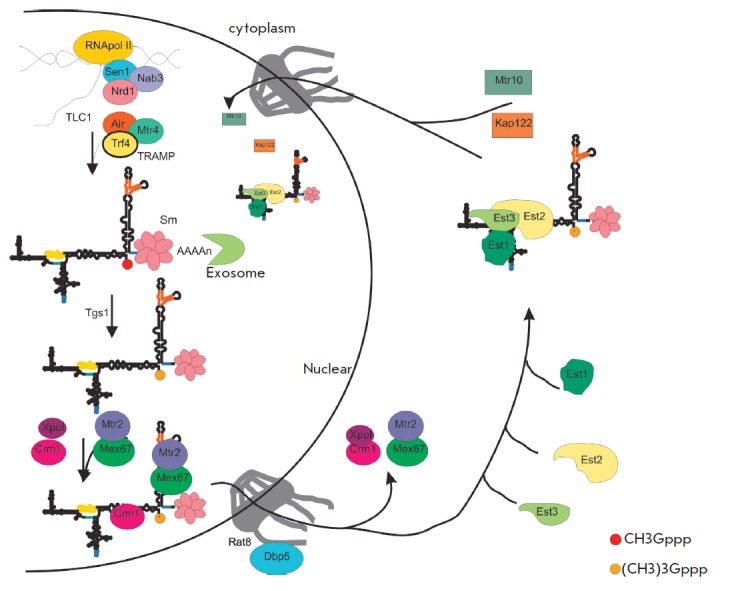
Model of processing and localization of *S. cerevisiae *telomerase RNA*. *

In yeast cells, different transcriptional complexes associated with RNA polymerase II participate in the formation of the two forms of the telomerase RNA primary transcript. At the transcription initiation step, RNA polymerase II forms a complex with termination and processing factors, i.e. the promoter determines the mechanism of termination. It has been demonstrated that the polyadenylated and non-polyadenylated forms of the primary transcript of *S. cerevisiae *telomerase RNA form independently. Disruption of polyadenylation signaling leads to the disappearance of the long polyadenylated form of TLC1, but it does not affect the formation of the non-polyadenylated mature form [18]. TLC1 is associated with Nrd1-Nab3-Sen1 transcription termination factors, specific for noncoding RNA [19]. The 3’-terminal region of the *TLC1 *gene contains binding sites for the termination factors Nab3 and Nrd1, whose deletion results in the accumulation of the polyadenylated primary transcript [18, 19]. The termination factors Nrd1, Nab3, and Sen1 are known to be associated with a complex consisting of RNA polymerase II, the cap-binding complex (CBP80, CBP20), an exosome, and TRAMP [20]. TRAMP includes TRF4/5 proteins (non-canonical poly(A) polymerase), Air1/2 (RNA-binding protein), and RNA-helicase MTR4 [21, 22]. TRF4 adds a short oligo(A) sequence, forming an unstructured 3’-terminus for noncoding RNAs as small nuclear, nucleolar, and TLC1, which can be processed by exosomes [18, 23, 24, 25, 26]. Exosomes activity is limited by the Sm-proteins associated with the 3’-terminal portion of mature telomerase RNA. If an exosome does not encounter an obstacle in its path in the form of a Sm-proteins complex, it fully degrades small nuclear and nucleolar RNA, as well as telomerase RNA [27] (*Fig. 2*).

**Cellular localization and assembly of the active telomerase complex of S.cerevisiae**

One of the important stages in the biogenesis of telomerase RNA and telomerase itself is the proper intracellular localization of their components (*Fig. 2*). As has been stated previously, the primary transcript of telomerase RNA in both yeast and human cells is subjected to co-transcription processing, followed by maturation or degradation by exosomes. In *S. cerevisiae, *properly matured telomerase RNA localizes in the nucleolus, where its cap is hypermethylated by the Tgs1 enzyme [28]. The trimethylated processed form of TLC1 is exported from the nucleus by a nuclear-cytoplasmic transport system [29]. The Crm1/Xpo1 and mRNA export factors Mex67 and Dbp5/Rat8 are responsible for the export of telomerase RNA. In the cytoplasm, telomerase RNA forms a complex with the protein subunits of telomerase Est1, Est2 and Est3, and afterwards the Mtr10 and Kap22 factors responsible for import into the nucleus transfer the enzyme back into the nucleus [29-31], where it interacts with telomeres and lengthens them in the late S-phase.

**Processing of telomerase RNA in fission yeast**

**Fig. 3:**
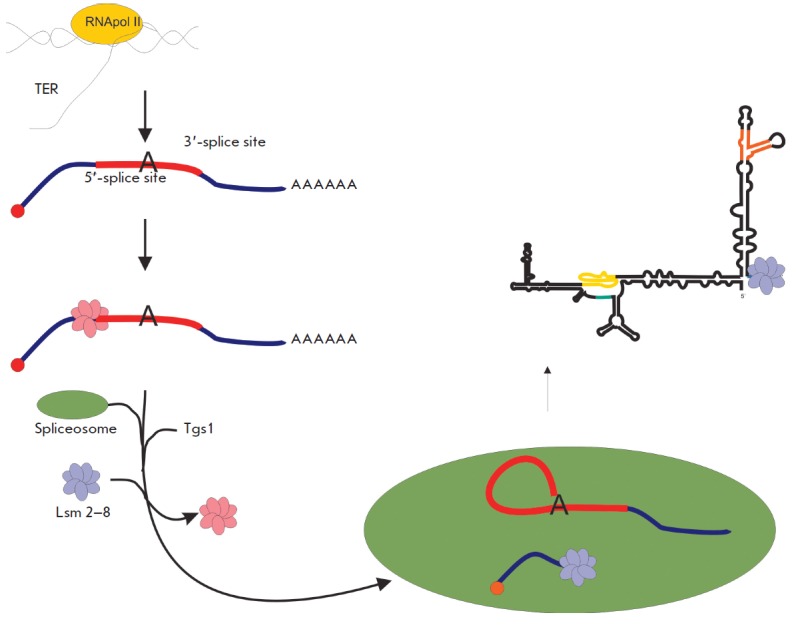
Model of processing of *S. pombe *telomerase RNA*.*

Telomerase RNA has undergone significant changes over the course of evolution, which have affected both its structure and processing mechanism. At the moment, there is no doubt that telomerase RNA is synthesized in all organisms as a long precursor whose correct processing results in mature catalytically active telomerase RNA. Telomerase RNA is involved in the fine regulation of the state of a cell; therefore, the content of telomerase RNA must be maintained at the physiological level for its proper functioning in a cell. In fission yeast (Schizosaccharomycetes) [32], *Hansenula polymorha *yeast (Saccharomycetaceae) [33], and other fungi (Sordariaceae, Trichocomaceae) [34], a precursor of telomerase RNA is synthesized by RNA polymerase II as a polyadenylated transcript (*Fig. 3*). The primary transcript of telomerase RNA in the cells of these organisms contains two exons, an intron, and a 3’-terminus poly(A) sequence. The processing of the primary transcript is carried out by a spliceosome. The first step in the splicing (cut in 5’-splicing site) produces a mature form of telomerase RNA. Processing by a spliceosome was first discovered in the cells of *Schizosaccharomyces pombe *yeast [32]. The splicing is a tightly coordinated process: all its stages occur very quickly and in a very specific order. In the first step, the 2’-hydroxyl group of adenosine at the branching point located in the middle of the branching site attacks the 5’-terminal splicing site at its sugar phosphate backbone. The result is an intermediate with a lasso structure, wherein the 5’-terminus of the intron is attached to the branching point through a 2’–5’-linkage. The freed 3’-hydroxyl group of the 5’-termnius of the exon attacks the 3’-terminal splicing site, which leads to a joining of exons and the excision of the intron as a lariat. The spliceosome contains small nuclear RNA (snRNA) U1, U2, U4, U5 and U6, which direct and enable a quick and accurate splicing process through complementary interactions with different parts of pre-mRNA [35]. It has been shown that a slowdown of TER precursor splicing after the effective first stage is caused by the peculiarities of the regulatory regions of telomerase RNA itself [32, 36]. In *S. pombe, *the distance between the branching point and 3’-terminal splicing site of telomerase RNA is 22 nucleotides [32], which is about two times greater than in the majority of introns in this organism [37]. Shortening the intron to 14 nucleotides leads to complete splicing and degradation of telomerase RNA [32]. Further analysis of the splicing sites has revealed some interesting features [31, 35]. It turns out that incomplete complementarity of the 5’-terminal splicing site of U1 snRNA [32], high degree of complementarity between the branching site and U2 snRNA, and a long distance between the branching point and the 3’-terminal splicing site, as well as weak polypyrimidine tract synergistically reduce the rate of transition to the second stage of splicing [36]. The PrP22 and PrP43 proteins (helicases with DExD/H-box) are involved in the processing of *S. pombe *telomerase RNA [36]. These proteins use the energy of ATP hydrolysis to release splicing intermediates during deceleration in the second stage (exon ligation). Therefore, when transition to the second stage of splicing is arduous, the spliceosomes frozen on intermediates are released [38]. The mutations inhibiting the ATPase activity of these proteins significantly increase the content of the fully spliced TER1 form [36].

Sm-proteins are known to be associated with the telomerase RNA of *S. cerevisiae *yeast [39], and they also interact with U1, U2, U4, and U5 snRNA [40, 41]. The Sm-proteins binding site is located a few nucleotides away from the 3’-terminus of the mature form [39]. In *S. pombe *cells, a spliceosome cuts TER1 at a distance of one nucleotide from the Sm-proteins binding site and therefore, may decrease the stability of the complex. It has been discovered that Sm-proteins interact with the polyadenylated TER1 precursor and facilitate its sectioning by the spliceosome [42]. Smd2 attracts Tgs1, which carries out post-transcriptional hypermethylation of TER1 to produce 2,2,7-trimethylguanozine 5’-cap. After the sectioning and hypermethylation of TER1, Sm-proteins dissociate and are replaced by Lsm-proteins (*Fig. 3*), which protect telomerase RNA against degradation by exosome [42].

Later, it was shown that other types of fission yeast and fungi maintain telomerase RNA processing by splicing its precursor with a spliceosome. In *S. cryophilius *and *S.octoporus, *the 5’-terminal splicing site comprises a cytosine residue in the third position, which stabilizes the interaction with U6 of snRNA in the first stage and slow transition to the second [43]. In *Aspergillus *sp. and *Neurospora crassa, *the first nucleotide of the 5’-terminal splicing site, adenine, is important for the release of the processed product after the first step of splicing [33, 42]. The formation of noncanonical interactions between the first and last guanosine in the intron is believed to be necessary for the positioning of the 3’-terminal splicing site in the second reaction of transesterification and ligation of exons [44], whereas replacement of guanosine with adenine in the telomerase RNA of the fungi families *Pezizomycotina *and *Taphrinomycotina*, which are the closest to the common ancestor, prevents the formation of a proper three-dimensional structure and stops the splicing after the first transesterification reaction and subsequent dissociation of the frozen spliceosome [34, 36, 43].

The dramatic differences in the mechanism of telomerase RNA processing in evolutionarily related organisms do not affect the strict control of the quantity and quality of the telomerase RNA in their cells. In yeast cells, exosome degrades improperly processed telomerase RNA as well as RNA which had not form complexes with the proteins that regulate its localization and activity.

**Processing and localization of human telomerase RNA**

Yeast and human telomerase RNA differ considerably in length and structure, but they share the main conservative components important for the formation and functioning of the telomerase complex. Mature human telomerase RNA (hTR) consists of 451 nucleotides [45]. Transcription of the *hTR *gene is carried out by RNA polymerase II [46]. The promoter of the *hTR *gene is well mapped, but the terminator region is poorly understood [47]. The length of the primary transcript of hTR remains to be determined. The 541-nucleotide elongated form of human telomerase RNA was first identified by reverse transcription, followed by PCR amplification [45]. More recent data obtained by high-throughput sequencing indicate the existence of a primary transcript of telomerase RNA up to 1,451 nucleotides in length [48]. The 3’-terminal domain of human telomerase RNA forms a structure similar to structures common to the H/ACA RNA family [49]. This structure consists of two hairpins connected by a single stranded loop H, and it contains a single stranded 5’-ACA-3 ‘motif located 3 nucleotides before from the 3’-terminus of the mature telomerase RNA (*Fig. 1*). H/ACA hairpins are associated with a set of four proteins: dyskerin, NHP2, NOP10, and GAR1 [13]. H/ACA hairpins and the proteins associated with it provide stability to telomerase RNA, as well as to other H/ACA RNA. It is known that H/ACA RNAs serve as guides for the directional pseudouridinylation of ribosomal RNA, but the telomerase RNA target is not defined: therefore, its H/ACA motif is assigned only a stabilizing function.

A combination of deep sequencing methods and determination of the 3’-terminus of RNA (3’-RACE) has revealed the heterogeneity of the 3’-terminus of human telomerase RNA [50]. It has been found that the 3’-terminal sequence may contain from one to seven additional nucleotides corresponding to the genomic sequence, and a short oligo(A) sequence (1–10 nucleotides). Thus, we can conclude that telomerase RNA is synthesized in the form of an elongated precursor which is processed to form the intermediate oligoadenylated form.

It is known that snRNAs containing H/ACA-motifs are processed by exosomes [51]. In mammalian cells, exosomes are attracted to their substrate by several protein complexes. The TRAMP (TRF4, ZCCHC7 and MTR4) complex is known to be involved in the degradation of noncoding RNAs and aberrant transcripts in the nucleolus. For this, the TRF4 protein oligoadenylates the transcript, which serves as a signal for degradation by exosomes [51, 52]. The NEXT complex (RBM7, ZCCHC8 and MTR4) attracts exosomes to actively transcribed RNA and the so-called PROMoter uPstream Transcripts (PROMPTs), whose synthesis begins before the coding genes promoters [53, 54]. NEXT interacts with the cap-binding complex (CBC), forming a CBCN complex and implementing co-transcriptional cap-dependent 3’-processing or degradation of RNA in the nucleus [54-57].

Inactivating mutations in the *PARN1 *gene encoding poly(A) ribonuclease 1 were recently discovered in patients with severe manifestations of dyskeratosis, a disease associated with short telomeres [58]. It was found that disruption of function or knockdown of the *PARN1 *gene leads to a decrease in the total amount of telomerase RNA in cells with a simultaneous increase in the proportion of non-processed oligoadenylated RNA [16, 47, 58, 59]. Baumann’s group discovered that spliceostatin A, a splicing inhibitor, does not affect the processing of telomerase RNA in humans [48], whereas isoginkgetin, which blocks the operation of not only spliceosome, but also exosomes [55], inhibits the processing of the hTR, resulting in the accumulation of the 3’-elongated form. In cells with a reduced content of the RRP40 protein, the main component of exosomes, and two nucleases associated with it, RRP6 and DIS3, the 3’-elongated form and the mature form of telomerase RNA accumulate, while the amount of the oligoadenylated form decreases. Mature telomerase RNA accumulates in the case of knocked down DGCR8 processor component, as well. It has been found that DGCR8 is involved in attracting exosomes to snRNA and telomerase RNA, thus controlling their total amount in a cell [60]. Knockdown of NEXT components, as well as that of the CBC complex promotes the accumulation of the 3’-elongated form of telomerase RNA [48]. The TRF4 protein, a component of TRAMP, and the canonical poly(A) polymerases PAPα and PAPγ carry out oligoadenylation of the telomerase RNA precursor [61]. Interestingly, oligoadenylation of telomerase RNA by the TRF4 protein promotes its degradation and PAPα/γ is involved in processing, which results in the formation of mature telomerase RNA [61]. The oligoadenylated form of human telomerase RNA is stabilized by PABPN1 (nuclear poly(A)-binding protein 1), which stimulates the synthesis of poly(A) sequences and attracts PARN, whereby promoting the maturation of hTR. A free oligo(A) sequence, unprotected by PABPN1, is a signal of RNA degradation by the TRAMP-exosome complex [61].

The proteins interacting with the H/ACA domain play a major role in the processing of human telomerase RNA. Dyskerin, NOP10, NHP2, NAF1, and GAR1 are RNA chaperones, and their interaction with telomerase RNA during processing stabilizes it, preventing degradation by exosomes. Dyskerin protects telomerase RNA from degradation by nuclear 3’-5’-exosomes. Dyskerin knockdown and mutations that disrupt telomerase RNA binding to the protein result in a reduced level of mature telomerase RNA in cells, whereas double knockdown of dyskerin and PARN1 cause the accumulation of telomerase RNA in cytoplasm bodies called cyTER (cytoplasmic TER). Degradation of telomerase RNA from the 5’-terminus by the decapping protein DCP2 and 5’-3’-exonuclease XRN1 [16] also indicate a cytoplasmic localization of telomerase RNA.

**Fig. 4:**
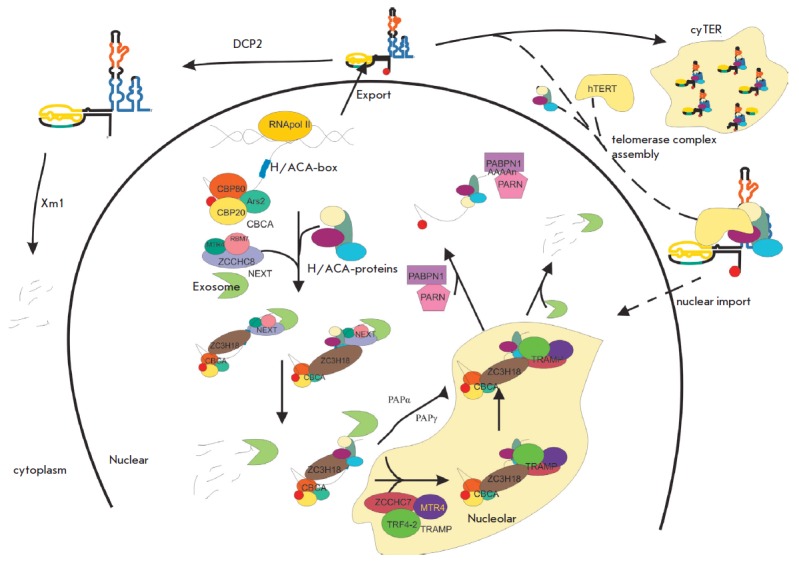
Model of processing and localization of human telomerase RNA*.*

Summarizing the data on the processing of human telomerase RNA, it is possible to suggest the following general scheme for its synthesis and maturation (*Fig. 4*). After primary transcript synthesis by RNA-polymerase II and co-transcriptionally capping, it interacts with dyskerin, NOP10, NHP2, and NAF1, which stabilize and protect RNA from degradation [62]. Part of telomerase RNA, which is associated with dyskerin and other chaperones, undergoes processing to form mature telomerase RNA. In order to achieve this, the CBC complex attracts the NEXT-exosome complex , which shortens the long precursor in the nucleus until it meets the H/ACA motif associated with H/ACA-binding proteins [48]. This product contains from one to seven additional nucleotides at the 3’-terminus. The nuclear poly(A) polymerases PAPα, PAPγ, and TRF4, a component of the exosome-associated nucleolar TRAMP complex, oligoadenylates this substrate [48, 60]. The oligoadenylated precursor interacts with PABPN1 [60], which protects it from further degradation, and attracts PARN1 [16, 48, 59, 60]. PARN1 gently shortens the oligo(A) sequence and the remaining additional nucleotides to form the mature telomerase RNA. The primary transcript which failed to form a complex with dyskerin or other chaperones is degraded by the TRAMP-exosome complex. Some of the primary transcript is exported from the nucleus to the cytoplasm, where it is decapped by the DCP2 protein and degraded by cytoplasmic 5’-3’-exonuclease XRN1 [16].

In a HeLa tumor cell line, telomerase RNA accumulates in Cajal bodies. A so-called CAB box is identified in the structure of telomerase RNA, which is responsible for its localization in the Cajal bodies, where telomerase and telomere interaction takes place [63]. Mutations in CAB-box [14], as well as mutations in the TCAB1 protein [64], disrupt human telomerase RNA localization in the Cajal bodies. TCAB1 interacts with CAB-box of telomerase RNA and ensures its location in the Cajal bodies [64]. Both the mutations and the absence of TCAB1 do not affect the enzymatic activity of telomerase, but they prevent its localization in Cajal bodies and telomeres [65]. hTERT may form a complex with hTR in both the nucleus and the cytoplasm, but the Cajal bodies and telomerase RNA direct telomerase localization at the telomere.

Recent research on the processing and localization of telomerase RNA in yeast and humans demonstrates that the amount of telomerase RNA in a cell is tightly controlled. Telomerase RNA processing and degradation are believed to be competing processes whose balance regulates the amount of telomerase RNA in a cell. The detection of telomerase RNA in the cytoplasm raises new questions. It is unclear whether this step is necessary for the processing or assembly of telomerase or whether human telomerase RNA performs alternative cell functions, some of which have been previously described [65].

## CONCLUSION

Telomerase maintains the proliferative potential of cells, which makes it one of the most important objects in studies of aging and cell transformation. Disruption of telomerase functioning leads to the development of tumors and telomeropathies. One of the essential components of telomerase is telomerase RNA, whose gene is expressed in most cell types throughout their lifetime. The expression of the *hTERT *gene which encodes the second component of telomerase is finely regulated, and the enzyme activation depends on the appearance of the hTERT protein in a cell. The mechanism of synthesis and processing of telomerase RNA has attracted the attention of scientists for more than 10 years, and recently there was a breakthrough in the study of this important stage of telomerase biogenesis. One of the most important features of telomerase RNA processing is the fine regulation of the content of this molecule in the cell. Both in yeast and human cells, telomerase RNA processing involves an exosome that rapidly degrades RNA that is not protected by RNA-chaperones. It has been established that most of the telomerase RNA gene transcription product is degraded in the process of biogenesis. Disruptions in the processing result in a degradation of the telomerase RNA that causes a number of diseases classified as telomeropathies.

Despite recent progress in understanding the mechanisms of telomerase RNA processing, there are questions to which we still have no answers. A full and detailed understanding of the mechanisms of both the functioning and biogenesis of telomerase will allow us to develop new approaches to the treatment of diseases whose development is associated with an impaired telomere maintenance system.

## Abbreviations

TER: telomerase RNA
hTR: human telomerase RNA
TLC1: S.cerevisiae telomerase RNA
TERT: telomerase reverse transcriptase
snRNA: small nuclear RNA
